# The Effects of Aging on Orientation Discrimination

**DOI:** 10.3389/fnagi.2017.00045

**Published:** 2017-03-02

**Authors:** Clara Casco, Michele Barollo, Giulio Contemori, Luca Battaglini

**Affiliations:** ^1^Department of General Psychology, University of PadovaPadova, Italy; ^2^Dipartimento di Beni Culturali, University of PadovaPadova, Italy

**Keywords:** vision, orientation, discrimination sensitivity, coding, encoding

## Abstract

Visual perception relies on low-level encoding of local orientation. Recent studies show an age-dependent impairment in orientation discrimination of stimuli embedded in external noise, suggesting that encoding of orientation is inefficient in older adults. In the present study we ask whether aging also reduces decoding, i.e., selecting the neural representations of target orientation while discarding those conflicting with it. We compared younger and older participants capability (mean age 24 and 68 years respectively) in discriminating whether the orientation of a Gabor target was left or right from the vertical. We measured (*d*′), an index of discrimination sensitivity, for orientation offset ranging from 1° to 12°. In the isolated target condition, *d*′ was reduced by aging and, in the older group, did not increase with orientation offset, thus resulting in a larger group difference at large than small orientation offsets from the vertical. Moreover, oriented elements in the background impaired more discrimination in the older group. However, distractors reduced more *d*′ when target-background orientation offset was large than when target and flanker had similar orientation, indicating that the effect of the background was not local, i.e., due to target inhibition by similarly oriented flankers. Altogether, these results indicate that aging reduces the efficiency in discarding the response to orientations differing from the target. Our results suggest that neural decision-making mechanisms, involving not only signal enhancement but also non-signal inhibition, become inefficient with age. This suggestion is consistent with the neurophysiological evidence of inefficient visual cortical inhibition in aging.

## Introduction

A well-documented finding in the psychophysical literature is that the perceptual system becomes less efficient with age (Owsley, 2011; Andersen, 2012). In addition to optical factors, neural factors play a role (Loerch et al., 2008). Inefficient inhibition has been associated to the age-dependent impairment in tasks involving center-surround neural suppression, such as orientation detection (Govenlock et al., 2009) and discrimination (Betts et al., 2007; Delahunt et al., 2008). In these studies, it was predicted that inefficient center-surround suppression could account for reduction in the ability of encoding^1^ orientation, on the basis of the hypothesis that it would reduce orientation selectivity of psychophysical channels. However, aging-dependent reduced inhibition may also affect decoding^2^ of the neural representation of target orientation because of suboptimal decision-related neural activity (Stone, 1960; Smith and Ratcliff, 2004; Gold and Shadlen, 2007; Wang, 2008; Deco et al., 2013), with consequent response to non-optimal orientation.

In human psychophysics, decision making in a binary orientation discrimination task requires a neural decoding operation (Gold and Ding, 2013) to select which of two alternative presented stimuli is the target. To decide whether the presented orientation is right or left from the vertical, the neural response to the presented orientation (positive) should be enhanced and that to the opposite (null) might be attenuated. The optimal response is to choose the population of orientation selective channels with the larger likelihood of responding to the presented target, but also to discard the alternative response (Deneve et al., 1999; Jazayeri and Movshon, 2006; Gold and Ding, 2013).

Monitoring and inhibition of psychophysical channels tuned to different orientations from the target is also involved when the target is embedded in a field of distractors differently oriented. Even in this condition of external noise, the visual system is faced with the problem of selecting the detectors activated by the target and discarding those activated by the distractors. In younger observers this task becomes easier as the difference in orientation between the target and background elements increases (e.g., Sagi and Julesz, 1987; Foster and Ward, 1991; Nothdurft, 1991; also Beck and Ambler, 1972; Bergen and Julesz, 1983; Foster and Westland, 1995). Age-dependent reduction of orientation selectivity would require larger target-background orientation contrast to equate the accuracy of the younger group. However, inefficient inhibition would impair performance even when target and background are highly discriminable, because background elements would also strongly activate orientation-tuned channels, which need to be inhibited to select the appropriate neural response.

Using Signal Detection Theory (SDT; Green and Swets, 1966) is appropriate for distinguishing how aging affects either sensitivity for orientation or the interpretation of neural response to orientation (Gold and Ding, 2013). In SDT, discriminability (*d*′) corresponds to the distance between the means of the two probability density functions with bell-shaped Gaussian distribution. The lower is such a distance as indexed by *d*′, the higher is the overlap of the two functions. Thus, if in an orientation discrimination task the two functions represent the tuning for either orientation to be discriminated, by calculating *d*′ it is possible to measure orientation discriminability over a range of orientation differences between the two putative targets.

For each orientation offset *d*′ calculation is based on two accuracy indexes: the correct responses for the presented orientation (hits) and incorrect responses for the non-presented orientation (false alarms, FA). Hits increase as the task becomes easier, i.e., when either target orientation offset from the vertical or target-background orientation offset increases. However, based on the assumption that detectors responding to both target orientations (and to distractors) are activated in each trial (Wang, 2008), reduced inhibition may prevent the correct choice to be made in the two-alterative forced choice (2AFC) task, thus discarding the response to the non-presented orientation (and to distractors). The consequence would be an increase of FA as the task becomes easier, instead of a decrease.

We measured *d*′ for discriminating target offset from the vertical ranging from 1° to 12°. The first prediction was that of an opposite outcomes on how younger and older adults would perform in the no noise condition, depending on whether the different performance between the two groups were accounted for by either inefficient encoding or inefficient decoding of orientation. A larger reduction of *d*′ in the older group at small than large orientation offset would be indicative of inefficient encoding of orientation by detectors inappropriately tuned to the oriented target. Oppositely, a larger reduction in *d*′ in the older group at large than a small target-non target orientation offsets would be indicative of inefficient decoding resulting from difficulty in discarding the irrelevant orientation. The second prediction was that oriented elements in the background (external noise) would have interfered with orientation discrimination in the two groups differently, depending on whether the difference in performance between the two groups was accounted for by either an encoding inefficiency or a decoding inefficiency in the older group. We predicted that inefficient encoding of orientation would have produced a larger group difference for the difficult task, i.e., when target and background elements had similar orientation. Conversely, we expected that inefficient decoding would have produced a larger group difference for the easy task, i.e., at larger orientation offset between target and distractors. In a second Experiment, group differences were assessed by opposing the role of background elements close and far from the target. Since close elements effect is more indicative of encoding inefficiency, we predicted larger group difference as resulting from age-dependent reduce efficiency in encoding orientation.

## Materials and Methods

### Observers

Two groups of 10 younger (11 males, 9 females) and of 15 older observers (16 males, 14 females) with normal or corrected to normal visual acuity participated. Each group participated in either in Experiment 1 or in Experiment 2. All subjects completed a questionnaire to screen for neurological and psychiatric disorders. All subjects participated voluntarily to the experiment and gave written informed consent according to the Declaration of Helsinki and the experimental methods have ethical approval from the University of Padova (protocol 1933). Full name of the ethics committee that approved the study: COMITATO ETICO DELLA RICERCA PSICOLOGICA AREA 17. For inclusion in the study, participants were either 21–30 years old (younger participants) or 65–75 years old (older participants). Documentation from their eye-care providers certified that the older subjects were free of strabismus, amblyopia, macular degeneration, cataracts and other ocular diseases. Mini Mental State Exam (MMSE) score ≥ 28 was also assessed (Folstein et al., 1975). Two potential older participants were excluded from the sample, one on the basis of the visual examination and the other on the basis of MMSE score.

### Stimulus

Participants were seated in a dark room 57 cm from the display screen. Viewing was binocular. The stimuli were created using Matlab and the Psychophysics Toolbox (Brainard, 1997; Pelli, 1997) and displayed on a 19-inch LCD Asus monitor with a refresh rate of 60 Hz. The screen resolution was 1920 × 1080 pixels. Each pixel subtended ~1.5 arcmin. A black fixation cross was presented at the center of the screen for 400 ms before the stimulus and remained present during stimulus presentation. The target: a Gabor (sinusoidal grating enveloped in a Gaussian window) that subtend 1° in size and with a spatial frequency of 2 cpd, appeared randomly at one of eight locations having the same eccentricity, for 33 ms.

We used the method of constant stimuli; in each trial the Gabor was tilted towards to the left or to the right from the vertical by one of six levels: 1°, 2°, 3°, 5°, 8° and 12°. The Gabor contrast was fixed (62% Michelson contrast, mean luminance 92 cd/m^2^). Observers perform a 2AFC orientation discrimination task; they judged whether the target was tilted either left or right from the vertical.

**Figure 1:**
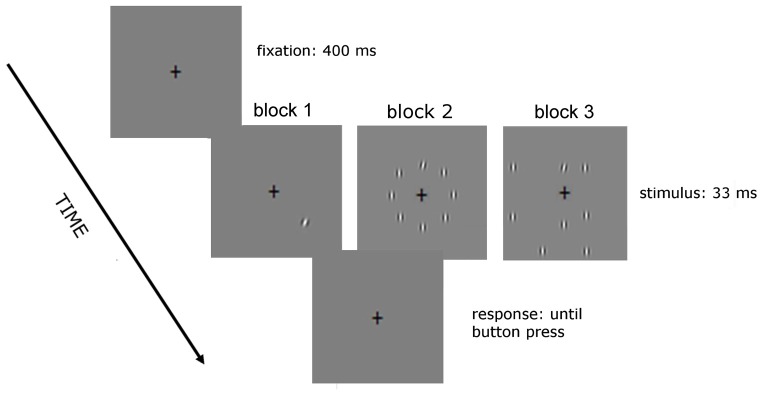
**Sequence of events in a given trial of Experiment 1.** Observers performed a two-alterative forced choice (2AFC) orientation discrimination task on a tilted target Gabor patch, which appeared at one of eight iso-eccentric locations for 33 ms. The fixation cross was presented before target presentation (for 400 ms) and during target presentation. We presented six different levels of orientation offset from the vertical using the method of constant stimuli. In the no noise condition only the target was presented, in the structured noise condition the noise elements were placed along an imaginary circle of 3.5° radius whereas in the random noise condition four out of seven elements were placed along the contour of an imaginary circle of 7° radius.

**Figure 2:**
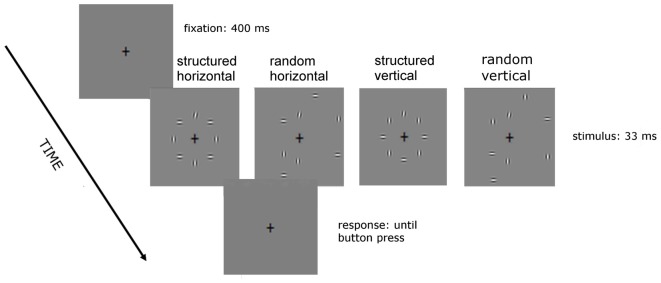
**Sequence of events in a given trial of Experiment 2.** The background elements position is as in Experiment 1 but their orientation is vertical and horizontal alternated, in such a way that the target is positioned either in between two horizontal flanking elements (as in the two left stimuli) or between two vertical elements (as between the two right stimuli).

The stimuli used are shown in Figures 1, 2. In the no-noise condition of Experiment 1 (Figure 1) only one target was presented, whereas in the noise condition there were seven noise elements vertically oriented, equally spaced and having the same size, spatial frequency and contrast as the target. In the structured noise condition, background and target elements were centered along an imaginary circle of 3.5° radius (structured noise). In the random noise conditions, four out of seven noise elements were centered along the contour of an imaginary circle of larger radius (7°; random noise).

In Experiment 2 (Figure 2), there were four noise conditions: structured horizontal, structured vertical, random horizontal and random vertical. They all consisted of vertical and horizontal elements alternated with either two vertical elements flanking the target, in the two vertical conditions of Figure 2; or two horizontal flankers, in the two horizontal ones. In the structured conditions, noise and target elements were centered along an imaginary circle of 3.5° radius. In the random conditions four out of seven noise element were centered along the contour of an imaginary circle of larger radius (7°). Note that although the position was not fixed, the high contrast of the target prevented for spatial uncertainty of target position.

### Procedure

For each group we used a within-subject design, with three blocks in Experiment 1 (no-noise, random noise and structured noise) and four blocks in Experiment 2 (structured horizontal, random horizontal, structured vertical and random vertical). Blocks presentation within each of the two experiment were counterbalanced. Within each block, the Gabor target orientation and location were randomly selected from trial to trial. Each lasted about 15 min and consisted of 96 trials resulting from the factorial combination of eight target positions, six target orientations and two directions of offset from the vertical.

### Data Analysis

We computed *d*′s, the distance between the mean of the two functions representing the tuning for either orientation to be discriminated. Observer’s sensitivity, *d*′, in units of standard deviation, is given by *d*′ = *z*(Hits)−*z*(FA) where Hits refer to saying “right (left)” when a rightwards (leftwards) oriented target was presented and FA refer to saying “right (left)” when a rightwards (leftwards) oriented target was not presented. Mixed-design analysis of variances (ANOVAs) with Group, Orientation and noise were used to evaluate as aging affects *d*′s obtained as a function of orientation offset. The Greenhouse-Geisser correction for degrees of freedom was applied here and in the following ANOVAs when appropriate, i.e., when the sphericity of the data was violated as indicated by a significant Mauchly’s test. *Post hoc* comparisons with Bonferroni correction were used for pairwise comparisons.

## Experiment 1: Age Differences in Orientation Discriminability with and without External Noise

In Experiment 1, we measure *d*′ as a function of orientation differences in no noise, structured and random noise conditions in the two groups of younger and older observers. We predicted that inefficient encoding in elderly would have likely manifested in a smaller *d*′ reduction in the older group at larger orientation offsets, where performance is less affected by either reduced selectivity for orientation (tuning) or reduced response to optimal orientation (gain). Conversely, if the group difference in *d*′ at large orientation offset was maintained, indicating that older observers did not take advantage from the easier task, then this would suggest inefficiency in decoding, i.e., inappropriately readout of the sensory information available after stimulus presentation, resulting in a smaller likelihood of the responding correctly to the presented stimulus. Furthermore, based on the inefficient decoding hypothesis, we expected that, when the target was presented with distractors, the group difference should have been more consistent when the target differed more in orientation from external noise elements. Furthermore, in younger observers we expected a major effect of background elements when their orientation was similar to the target (Foster and Westland, 1995). Since age differences may depend on target and background elements’ positional arrangement (Casco et al., 2011, 2012), we used two types of noise: random and structured, i.e., arranged along a virtual contour.

### Results

**Figure 3:**
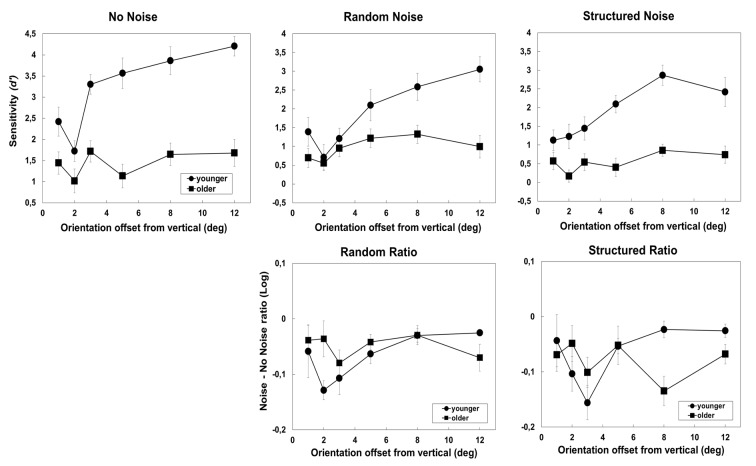
**Results obtained in the no-noise and 1-orientation noise condition of Experiment 1.** The left panel shows the result obtained in the no-noise condition, the central panels show the results in the random condition, and the right panels show the results obtained in the structured condition. The three top panels show sensitivity (*d*′) as a function of orientation offset from the vertical. The two bottom panels show the effect of noise—expressed as log_10_(Noise/No-Noise)–as a function of orientation offset in the two groups. Error bars ± SEM.

The effect of external noise is shown in Figure 3 for no noise (left) random (center) and structured noise (right) respectively. The top panels show *d*′s as a function of orientation offset in the two groups. The graphs clearly show a larger sensitivity difference amongst the two groups when the task is easy in both noise and no noise conditions. Moreover, in the older group, *d*′s were lower in the structured than random noise condition.

The mixed-ANOVA with Group, Noise type (no-noise, structured and random) and Orientation levels as factors revealed a significant effect of Group (*F*_(1,23)_ = 50.03, *p* < 0.0001, *partial-η*^2^ = 0.69), Noise (*F*_(2,46)_ = 54, *p* < 0.001, *partial-η*^2^ = 0.7) and Orientation (*F*_(5,115)_ = 18.13, *p* = 0.001, *partial-η*^2^ = 0.44). The interaction of Group × Orientation was significant (*F*_(5,115)_ = 7.73, *p* < 0.001, *partial-η*^2^ = 0.25). *Post hoc* comparisons (Bonferroni correction) revealed that, in the younger group, *d*′ generally increase with orientation offset except for the two lowest orientation offsets that did not present significant differences (*p* = 1). In addition, the group difference was significant at 2 (*p* < 0.01), 3 (*p* < 0.01), 5 (*p* < 0.001), 8 (*p* < 0.001) and 12° (*p* < 0.001) but not at 1° orientation offset (*p* = 0.18). The interaction Group × Noise type was also significant (*F*_(2,46)_ = 7.1, *p* = 0.002, *partial-η*^2^ = 0.24), indicating that in the older group only, the sensitivity (*d*′) was significantly higher in the random noise compared to the structured noise condition (*p* = 0.017). None of the other interactions were significant: noise × orientation (*F*_(10,230)_ = 1.1, *p* = 0.33, *partial-η*^2^ = 0.047) and group × noise × orientation (*F*_(10,230)_ = 1.06, *p* = 0.39, *partial-η*^2^ = 0.044).

The lower panels of Figure 3 show that the effect of noise—expressed as log_10_(Noise/No-Noise)—was larger in the older group at larger orientation offsets. The ANOVA with group, noise type and orientation levels as factors revealed a significant effect of noise (*F*_(1,23)_ = 6.7, *p* = 0.016, *partial-η*^2^ = 0.23), orientation (*F*_(1,23)_ = 3.2, *p* = 0.01, *partial-η*^2^ = 0.12) and of the interaction of group × orientation offset (*F*_(5,115)_ = 3.5, *p* = 0.005, *partial-η*^2^ = 0.13). *Post hoc* comparisons (Bonferroni correction) showed a higher effect of noise in younger than in the older observers at 2° (*p* = 0.03) when the task was difficult and higher effect of noise in older observers than in the younger group at 8° (0.018) and almost at 12° (*p* = 0.06) when the task was easier. The pairwise comparisons, across orientations revealed that, in the younger group, noise had more detrimental effect at 2° and 3° orientation offset than at 8° (*p* = 0.03, *p* = 0.013) and 12° (*p* = 0.016; *p* = 0.029) whereas for the older groups the effect of noise did not differ amongst orientations. The lower panels of Figure 3 show a clear increase, in the younger group, of the noise effect when orientation offset increases from 1° to 3°, a results reflecting that an increase in orientation offset within this range progressively increase discriminability in the no noise more than in the noise condition.

## Experiment 2: Age-Dependent Effect of External Noise Type

The evidence that the effect of noise depends on age opens an interesting question: is this age effect dependent on the orientation offset between the target and the background elements flanking the target? Likely mediated by short-range inhibitory connections (Das and Gilbert, 1999), iso-oriented elements flanking the target inhibit the target itself. Orientation offset reduces this surround suppression (Knierim and van Essen, 1992; Lamme, 1995; Kastner et al., 1997; Caputo and Casco, 1999; Akasaki et al., 2002; Giora and Casco, 2007; Casco et al., 2009; Alberti et al., 2010). This local target-flanker inhibition impairs the formation of a neural representation for the incoming input. If the impairment was larger for older adults, thus age differences in *d*′ should be larger when the target and near flanker have similar with respect to when they have different orientation.

To address the issue of the role of local lateral intra-cortical inhibition in aging, we designed a new experiment in which participants had to discriminate the orientation in two flanking orientation conditions: either similar or dissimilar to the target. To this end we used a background with mixed orientations, vertical and horizontal alternated, either random or structured (Figure 2). Although in both conditions elements were similarly interleaved, in one condition the flankers had vertical orientation, that is similar to that of the target (i.e., 1°–12° offset vertical), whereas in the other condition flanker orientation was horizontal, thus dissimilar to that of the target.

### Results

**Figure 4:**
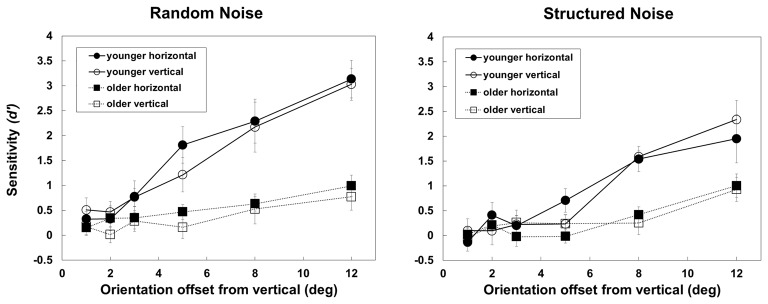
**The figure shows *d*′s obtained by younger and older observers in the two background-structure conditions (random, on the left panel, and structured, in the right panel) with the two background elements flanking the target are horizontal (thus, with relatively high orientation offset with the target) or vertical (with orientation similar to the target).** Error bars ± SEM.

Figure 4 shows, separately for the random and structured noise conditions, *d*′s obtained by the two groups when the orientation of background elements flanking the target was either similar to the target or not similar. From the figure it is clear that aging reduces *d*′s in a similar way in the two flanking conditions, i.e., more consistently at higher orientation offsets.

Indeed, the three-way mixed-design ANOVAs on *d*′s revealed a significant effect of Group (*F*_(1,23)_ = 17.59, *p* < 0.001, *partial-η*^2^ = 0.43), Noise (*F*_(1,23)_ = 24.73, *p* < 0.001, *partial-η*^2^ = 0.518), Target orientation (*F*_(5,115)_ = 56.3, *p* < 0.001, *partial-η*^2^ = 0.71) but not of Flaker orientation (*F*_(1,18)_ = 1.017, *p* = 0.32, *partial-η*^2^ = 0.042). The Group × Noise (*F*_(1,23)_ = 11.5, *p* < 0.001, *partial-η*^2^ = 0.33) and Group × Target orientation interaction were also significant (*F*_(5,115)_ = 12.26, *p* < 0.001, *partial-η*^2^ = 0.42). *Post hoc* comparisons (Bonferroni correction) revealed a significant effect of Group with random (*p* < 0.001) and structured noise (*p* = 0.005) and a significant reduction in performance with structured noise in the younger (*p* < 0.001) but not older group (*p* = 0.225); moreover there was an effect of group at 5 (*p* < 0.001), 8 (*p* < 0.001) and 12° offset (*p* < 0.001) only. None of the other interactions was significant.

## General Discussion

Although *d*′ at the two smallest orientation offsets of the isolated target was relatively high in both groups (average correct probability ≥70%), indicating that both groups could perform the task, it did not increase with orientation offset in the older group. Thus, the two groups mostly differ at large orientation offset from the vertical, when orientation discrimination task was easier. Moreover, results showed a lower effect of noise at large than small orientation offsets in the younger but not older group. Experiment 2 showed that group difference was not affected by whether the target orientation was similar or different to that of flanking background elements.

### The Effect of Aging on Orientation Discrimination

It is worth discussing whether the age effect in the no-noise is consistent with the evidence that older subjects have inefficient response to orientation (Betts et al., 2007) which may be accounted for by reduced contrast sensitivity (Pardhan et al., 1996; Bennett et al., 1999; Delahunt et al., 2008). Our results do not exclude the possibility that, if tested in appropriate conditions, older participants would have shown reduced selectivity for orientation (tuning) or reduced response to optimal orientation (gain; Jazayeri and Movshon, 2006; Betts et al., 2007). These outcomes are predicted on the basis of inefficient encoding resulting from reduced surround inhibition (Tadin and Blake, 2005; Casco et al., 2015) and consequent changes in the inhibitory/excitatory balance of neural response (Freret et al., 2015; Fu et al., 2015). There is however no general consensus in previous studies on the inefficient encoding hypothesis. Some found age differences in orientation selectivity (tuning) of the underlying neural detectors, suggesting that the tuning of orientation sensitive cortical cells is reduced in the aged human visual cortex (Betts et al., 2007). Others, using either interval-2AFC detection (Govenlock et al., 2009) or orientation discrimination tasks (Delahunt et al., 2008) did not replicate this result, suggesting that differences in contrast detection sensitivity account for group differences.

Without excluding reduced sensitivity for contrast and orientation in older subjects under appropriate conditions, this impairment does not account for the result that *d*′ was reduced more in the older group when the task was easier. This suprathreshold age effect is more compatible with the hypothesis that older observers have difficulty in inhibiting irrelevant orientations, i.e., the null orientation in the no-noise condition (Deneve et al., 1999; Jazayeri and Movshon, 2006). Indeed, previous studies showed that as decision becomes more dependent on psychophysical channels responding inappropriately to target orientation, the function relating *d*′ to orientation offset shifts rightwards (indicating higher threshold) and have lower values for high stimulus strength (Gold and Ding, 2013).

### The Age-Effect Effect on Orientation Discrimination with External Noise

The effect of external orientation noise in Experiment 1 seems more consistent with the hypothesis that reduced inhibition makes neural decision inefficient rather than promoting a compensatory neural response to impoverished sensory input (Stothart et al., 2013). Indeed, the presence of external noise, in particular when structured, impaired more the older group but when the task was easy, indicating that the effect of noise decreased as orientation offset increased in the younger but not older group. This result together with the evidence of Experiment 2, that reducing the orientation similarity between target and flanker, a variable that affects encoding, does not improves discrimination in older adults, supports the decoding hypothesis. Difficulty in decoding may arise from higher dependency on psychophysical channels inappropriately tuned to orientation when their response is not discarded: either those activated by the null orientation of the putative target or those activated by the background orientations (Serences and Boynton, 2007; Boynton, 2009).

In the present study, we assumed that SDT successfully capture decision on which of two alternative has to be selected on the basis of a single observation, with or without external noise present. Indeed, this basic linear conceptual model predicts that an increase of orientation offset leads either to decrease of FA, as evidence of facilitated encoding, or to an increase of FA, as reflecting higher interference from the irrelevant orientation/s in the decision process. However, it is worth discussing the implementation of this model in a physiological plausible way, in order to relate the effect of aging in orientation discrimination to a specific feature of the decision computation process.

### The Effect of Aging-Dependent Inhibition on Orientation Discrimination

A physiologically realistic candidate model for decision making (Wang, 2008) assume that one distinguishing feature of decision making consists of a neural mechanism for temporal accumulation of evidence in favor or against alternative input signals. In a 2AFC task, temporal accumulation first occurs by the activation, in each trial, of two subpopulations of detectors each selective for either of the two putative target orientations. Age-dependent downregulation of excitatory neural response or increase of internal noise may decrease the recurrent facilitation within detectors in each subpopulation, thus reducing the difference in activation of detectors responding to the presented and null orientation (Deco et al., 2013); however, oppositely to what we found, the model predicts that a sufficient difference in activation should remain at large orientation offset. The other distinguishing feature of the model is the competitive feedback inhibition between the two subpopulation of detectors for the formation of a categorical choice. The model assumes that competitive inhibition occurs by the GABA-dependent activation of a third subpopulation of inhibitory neurons. If competitive inhibition became inefficient with age, due to a downregulation of GABA response, this could account for the result that orientation discrimination did not improve with orientation offset in older observers, a result consistent with inefficient decision making. There is neurophysiological evidence that visual cortical inhibition decreases with age (Leventhal et al., 2003). These authors found that response to orientation becomes more selective by local administration of GABA and GABA agonists, suggesting that reduced GABAergic functioning in the older brain could be the underlying mechanism for reduced inhibition. Human data indicated that GABA system, which mediates inhibitory neurotransmission, may be downregulated in the aging prefrontal cortex (Loerch et al., 2008), and area considered as a good neural candidate, together with the parietal cortex, for decision computation.

In conclusion, the evidence that GABA is involved not only in coding of orientation by mediating orientation tuning by center-surround suppression but also in decision-making, by mediating winner-takes-all competition in neural decision (Wang, 2008; Deco et al., 2013), together with the evidence of age-dependent GABA downregulation, could explain the present result of reduced orientation discrimination in easy conditions in older adults.

## Author Contributions

CC: study conception and design. MB, LB and GC: acquisition of data. CC, LB, MB and GC: drafting of manuscript.

## Funding

This research was supported by grants to CC from University of Padova.

## Conflict of Interest Statement

The authors declare that the research was conducted in the absence of any commercial or financial relationships that could be construed as a potential conflict of interest.

## Footnotes

^1^Encoding: extract sensory information in the brain.

^2^Decoding: readout the extracted sensory information in the brain in order to form a decision variable, which applied to a decision rule, determines the final choice (Graham, 1989).
